# 6′-Hydroxy Justicidin B Triggers a Critical Imbalance in Ca^2+^ Homeostasis and Mitochondrion-Dependent Cell Death in Human Leukemia K562 Cells

**DOI:** 10.3389/fphar.2018.00601

**Published:** 2018-06-06

**Authors:** Jiaoyang Luo, Jiaan Qin, Yanwei Fu, Shanshan Zhang, Xingguo Zhang, Meihua Yang

**Affiliations:** ^1^Key Laboratory of Bioactive Substances and Resources Utilization of Chinese Herbal Medicine, Ministry of Education, Institute of Medicinal Plant Development, Chinese Academy of Medical Sciences and Peking Union Medical College, Beijing, China; ^2^School of Life Sciences and Engineering, Southwest Jiaotong University, Chengdu, China

**Keywords:** 6′-hydroxy justicidin B, mitochondria, Ca^2+^ homeostasis, caspase, apoptosis, pharmacokinetics

## Abstract

*Justicia procumbens* (*J. procumbens*) is a traditional Chinese herbal medicine which was used for the treatment of fever, pain, and cancer. A compound 6′-hydroxy justicidin B (HJB) isolated from *J. procumbens* exhibits promising biological properties. However, the mechanism of action and the *in vivo* behavior of HJB remain to be elucidated. In this study, we investigated the mechanism of action of HJB on human leukemia K562 cells and its pharmacokinetic properties in rats. The results demonstrated that HJB significantly inhibited the proliferation of K562 cells and promoted apoptosis. Besides, HJB resulted in decreased mitochondrial membrane potential deltaPSIm, increased the level of the calcium homeostasis regulator protein TRPC6 and cytosolic calcium. The activity of caspase-8, caspase-9 and the expression of p53 were significantly increased after treatment with HJB. Additionally, HJB has rapid absorption rate and relative long elimination *t*_1/2_, indicating a longer residence time *in vivo*. The results indicate that HJB inhibited the proliferation of K562 cells and induced apoptosis by affecting the function of mitochondria and calcium homeostasis to activate the p53 signaling pathway. The pharmacokinetic study of HJB suggested it is absorbed well and has moderate metabolism *in vivo*. These results present HJB as a potential novel alternative to standard human leukemia therapies.

## Introduction

With chemotherapy remaining the popular cancer treatment, research and development of anti-tumor drugs has been a hotspot in recent years (Kerru et al., 2017). Anti-cancer drugs such as imatinib and dasatinib, which were designed for specific cancers, often yield favorable outcomes. However, drawbacks including acquired drug resistance and serious side effects have hindered their application (Schlemmer et al., 2011; Uzer et al., 2013; Wu et al., 2017). Therefore, development of efficient anti-tumor drugs with low toxicity is still urgently needed.

Studies have shown that justicidins have a variety of biological activities, such as neuroprotection (Gu et al., 2016), cytotoxicity (Hemmati and Seradj, 2016), antiviral activity (Asano et al., 1996), and a wide range of anti-tumor effects. As a potential bioactive substance, its cytotoxicity to numerous cells lines has been systematically studied (He et al., 2012; Ilieva et al., 2014; Jin et al., 2014; Momekov et al., 2014; Won et al., 2015). 6′-hydroxy justicidin A (HJA) was reported to induce apoptosis through the caspase pathway (He et al., 2012). However, the associated mechanism of 6′-hydroxy justicidin B (HJB) as a potential antitumor compound remains unknown.

Mitochondria are essential for cellular maintenance of normal oxidative phosphorylation, and play an important role in apoptosis control (Cadenas, 2004). Mitochondrial membrane potential (Δψm) is required for normal mitochondrial function, and changes in Δψm can be an indicator of apoptosis. The mitochondrial damage caused by the chemical imbalance between the cytoplasm and mitochondrial matrix leads to mitochondrial intimal rupture, releasing cytochrome c, apoptosis induced factor (AIF), and procaspase (Korge and Weiss, 1999; Bernardi et al., 2006; Leytin et al., 2009). The caspase family plays an essential role in the signal transduction and effector processes of apoptosis (Earnshaw et al., 1999; Los and Wesselborg, 1999; Nicholson, 1999; Boatright and Salvesen, 2003; Schwerk and Schulze-Osthoff, 2003). Ca^2+^ acts as a second messenger, causing apoptosis through its effects on mitochondrial function and other relevant pathways (Bhandary et al., 2012). The increase of cytoplasmic Ca^2+^ concentration is also an early signal of apoptosis. p53 plays an important role in carcinogenesis, and has been a frequent target in recent years (Batchelor et al., 2011; Purvis et al., 2012; Paek et al., 2016). Related reports indicate that p53 induces apoptosis by directly affecting the structure and function of mitochondria (Matoba et al., 2006).

Preliminary studies investigating the effect of HJB on apoptosis in K562 cells, indicated that HJB could decrease SOD activity and increase of caspase-3 activity in K562 cells (Luo et al., 2014a). The cytotoxicity of HJB to different tumor cell lines was measured, including MDA-MB-157, HepG2, MGC-803, and K562. Compared with other cell lines, HJB had the lowest inhibition concentration (IC_50_) for K562 cells. Therefore, in the present study, the K562 cell line was used to study the effect of HJB treatment on Δψm, calcium homeostasis, caspase-8, -9 activity and p53 expression. In addition, we developed a sensitive and accurate UFLC-ESI-MS/MS method for quantification and pharmacokinetic study for HJB after its oral administration in rats.

## Materials and Methods

### Materials and Reagents

Imatinib (IMA) with purity of more than 99% was purchased from Beijing Century Aoke Biological Technology Co., Ltd. HJB was isolated from *J. procumbens* and identified by UV, IR, and ^1^H and ^13^C-NMR (Yang et al., 2006). Buspirone (Internal standard, IS, batch No. 039K1325) was purchased from Sigma (St. Louis, MO, United States). The other relevant materials and reagents are listed in Supplementary Materials: Text 1.

### Equipment and Chromatographic-Mass Spectrometric Conditions

The UFLC system consisted of two LC-20AD pumps, an SIL-20AC autosampler, a CTO-20A column oven (Shimadzu, Japan) and a CBM-20A controller. The UFLC separation was performed on an Agilent 300SB-C_18_ column (2.1 mm × 50 mm, 3.5 μm). The UFLC system was coupled with a 5500 QTRAP mass spectrometer (Applied Biosystems/MDS Sciex, Concord, ON, Canada) via a Turbo IonSpray ionization interface. The chromatographic-mass spectrometric conditions are described in Supplementary Materials: Text 2, 3.

### Animals

Wistar rats, male, weighing 180–220 g, were obtained from Beijing HFK Bioscience Co., Ltd. (License No. SYXK 2009-0007). The animals were raised separately by gender and had unlimited access to food and water in an environmentally controlled breeding room (temperature 22 ± 2°C, humidity 60–80%). The breeding room was illuminated by an artificial light cycle with 12 h of light and 12 h of darkness every day, and it was disinfected regularly.

### Cell Cultures

Human hepatocellular carcinoma cell line (HepG2), human breast cancer cell line (MDA-MB-157), human gastric cancer cell line (MGC-803), human promyelocytic leukemia cell line (HL60) and healthy macrophage cell line RAW 264.7 were obtained from the Cancer Institute and Hospital, Chinese Academy of Medical Sciences. Human leukemia cell line (K562) was obtained from the Institute of Basic Medical Sciences of the Chinese Academy of Medical Sciences. The detailed protocol of cell culture is described in Supplementary Materials: Text 4.

### Cell Viability Assay

Cytotoxicity was measured by a modified MTT assay (Luo et al., 2014a). Cells were treated with different concentrations of HJB (0.89, 7.24, 14.44, 28.89, 57.80, and 115.60 μM) for 48 h, and the cytotoxicity was measured by MTT assay. IMA was used as a positive drug to investigate the cytotoxicity of different concentrations (0.04, 0.26, 0.53, 1.05, 2.09, and 4.17 μM) on K562, HepG2, MDA-MB-157, and MGC-803 cell lines. In addition, the cytotoxicity of HJB on HL60 and RAW 264.7 were investigated. Each assay was carried out in triplicate. The detailed protocol of modified MTT assay is described in Supplementary Materials: Text 5.

### Annexin V/PI Staining and Flow Cytometry Analysis

Annexin V-FITC is highly affinity bound to the extracellular membrane of PS, used to label early apoptotic cells. Propidium iodide (PI) is a nucleic acid dye that can pass through the cell membrane of late apoptotic cells and bind to nucleic acids to label late apoptotic cells. Annexin V-FITC was used in combination with PI, and the stained cells were analyzed by flow cytometry. In the present study, the apoptosis of K562 cells treated with HJB was analyzed by the Annexin V-FITC Kit. The experiment was carried out in triplicate. The detailed protocol of Annexin V/PI staining and flow cytometry analysis is described in Supplementary Materials: Text 6.

### Detection of Δψm

JC-1 (5,5′,6,6′-tetrachloro-1,1′,3,3′-tetraethylbenzimidazolylcar-bocyanine iodide) is a fluorescent probe for detecting mitochondrial membrane potential. At normal mitochondrial membrane potentials, JC-1 can aggregate in the mitochondrial matrix to form a polymer that exhibits red fluorescence. When the mitochondrial membrane potential becomes lower, the JC-1 will keep monomer that exhibit green fluorescence. Changes in mitochondrial membrane potential could be delineated by the fluorescence. In the present study, the Δψm was detected by fluorescence microscopy using the JC-1 mitochondrial membrane potential assay kit as described in Supplementary Materials: Text 7. The experiment was carried out in triplicate. The fluorescence intensity was analyzed by Image-Pro Plus 6.0 software, and the ratio red vs. green fluorescence was calculated.

### Ca^2+^ Homeostasis Assay

Fluo 3-AM is an acetyl methyl ester derivative of Fluo 3, which is a commonly used fluorescent probe for detecting intracellular Ca^2+^ concentration. Fluo 3-AM enters the cell and is cleaved by intracellular esterase to form Fluo-3, which is retained in the cell. Fluorescence-free Fluo-3 binds to intracellular Ca^2+^ to produce strong fluorescence for display intracellular calcium distribution. The Ca^2+^ homeostasis assay was performed by fluorescence microscopy as described in Supplementary Materials: Text 8. The experiment was carried out in triplicate, and the fluorescence intensity was analyzed by Image-Pro Plus 6.0 software.

### SERCA and TRPC6 Expression

The sarco (endo) plasmic reticulum Ca^2+^-ATPase (SERCA) pumps actively transport Ca^2+^ across the sarcoplasmic reticulum (SR) membrane from the cytosol and into the SR and play a primary role in the maintenance of a large cytosolic – SR Ca^2+^ gradient (Gamu et al., 2014). Transient receptor potential (TRP) channels form a superfamily of non-selective cation channels that widely function as cell multi-signal transducers, and that sustained TRP canonical 6 (TRPC6) plays an important role in the regulation of calcium homeostasis in cell signaling (Rowell et al., 2010). The surface expression level of TRPC6 in cell extracts and SERCA released in supernatants of cell culture were measured by ELISA using enzyme-linked immunosorbent assay kits in accordance with the manufacturer’s protocol. All the experiments were carried out in triplicate.

### Caspase-8 and Caspase-9 Activity Assay

Caspase-8 is a key mediator of apoptotic signals triggered by death receptors, but inter-chain cleavage of caspase-8 in the mitochondrial pathway is sufficient to produce an active enzyme even in the absence of receptor-driven procaspase-8 dimerization during drug-induced apoptosis (Sohn et al., 2005). The activity of caspase 8 and caspase 9 in cell was measured by spectrophotometry as described in Supplementary Materials: Text 9. All the measurements were carried out in triplicate.

### Expression of Cytochrome c in Mitochondria and Cytosol of K562 Cells

To assess cytochrome c release from mitochondria, the mitochondria and cytosol were isolated using a cell mitochondria isolation kit (Beyotime Biotech Inc., China). The isolation process was referred to a previous literature (Yang et al., 2017). Cells were rinsed and harvested in a lysis buffer. Protein concentrations were determined by BCA protein assay kit. The cytosolic (supernatant) and mitochondrial fractions (pellet) were separated by SDS-PAGE, and gel was transferred to PVDF membranes. Proteins were detected through antibody to cytochrome c. Quantitative analysis of the blots was performed with an imaging densitometer. The experiment was carried out in triplicate.

### p53 Expression Assay

The p53 protein in cells was labeled with Anti-p53 pS37-PE, and the expression of p53 was detected by flow cytometry. REA Control (I) antibodies, an antibody from the same species as Anti-p53 pS37-PE was used as an isotype control to eliminate the background staining of Anti-p53 pS37-PE against non-specific adsorption of cells. The detailed protocol of the p53 expression assay is found in Supplementary Materials: Text 10. The experiment was carried out in triplicate.

### Preparation of Standards and Calibration Curves

Separate stock solutions of HJB and IS (1.0 mg/mL) were prepared by dissolving appropriate amount of each reference standard in DMSO, and were refrigerated until used. A series of the working standard solutions were prepared by appropriate dilutions of the stock standard solutions with DMSO. These standard solutions were used to spike blank rat plasma to yield calibration standards. The preparation of HJB, IS standards and quality control samples were performed as described in Supplementary Materials: Text 11.

### Sample Treatment of Plasma Samples

Aliquots of 50 μL rat plasma were mixed with 5 μL of methanol and 10 μL of IS solutions and 400 μL of ethyl acetate. After vortex for 1 min and then centrifugation at 13,000 × *g* for 10 min, aliquots of 300 μL supernatants were removed and evaporated to dryness at 40°C under a gentle stream of nitrogen. The residues were dissolved in 100 μL of the mixture of methanol and water (50:50, *v/v*), and 5 μL of this solution was then injected onto the column.

### Pharmacokinetic Study in Rats

This study was carried out in accordance with the recommendations of the Institutional Animal Care and Use Guidelines in China. The protocols were approved by the Institutional Animal Care of Medicinal Plant Development, Chinese Academy of Medical Sciences (SLXD-2017042319). Six rats were divided into three groups at random (*n* = 3 rats/group). All experimental procedures were approved by the Experimental Animal Care and Use Committee of Peking Union Medical College (Beijing, China). The detailed protocol is found in Supplementary Materials: Text 12.

### Statistical Analysis

All experimental data are shown as means ± SD and accompanied by the number of experiments. Analysis was performed using one-way ANOVA followed by Dunnetts *post hoc* test, and the values for significant difference were set at ^∗^*p* < 0.05, ^∗∗^*p* < 0.01.

## Results

### Comparison of the Cytotoxicity of HJB Among Different Cell Lines

**FIGURE 1:**
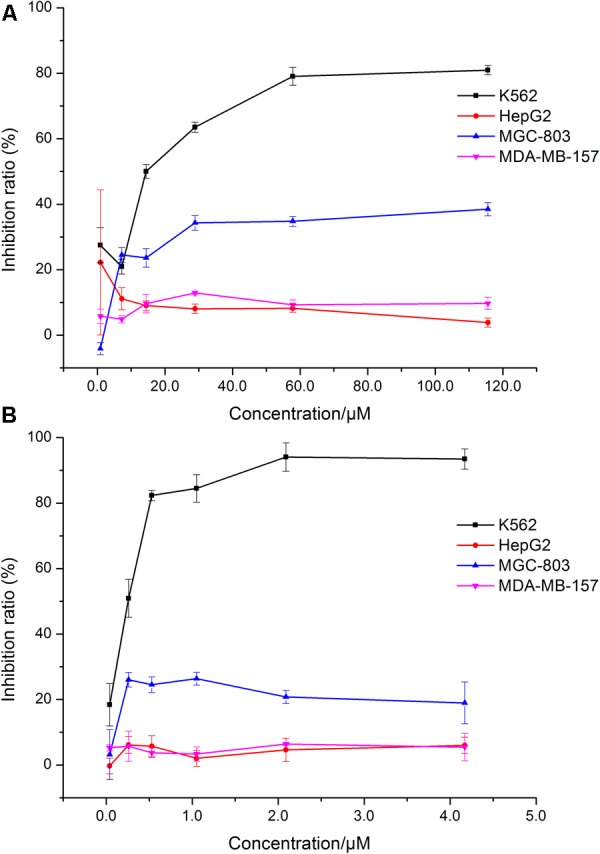
Comparison of the effects of HJB and IMA on different cell lines. **(A)** Effect of HJB on K562, HepG2, MGC-803, and MDA-MB-157 cell proliferation. **(B)** Effect of IMA on K562, HepG2, MGC-803, and MDA-MB-157 cell proliferation. Data represented the mean ± SD of three independent experiments, where each sample was tested in at least triplicate.

In this test, we designed to screen the cytotoxicity of HJB on cancer cells and normal cells. The dose-effect curve of HJB and IMA for different cell lines is shown in **Figure 1**. The IC_50_ of HJB as measured by MTT assay was 15.07 ± 1.01 μM for K562 cell line (**Figure 1A**) in accordance with a previous report (20.0 ± 1.7 μM) (Luo et al., 2014a). Compared with other cell lines (HepG2, MDA-MB-157, and MGC-803 cell lines), HJB had stronger cytotoxic effects on K562 cells. The IC_50_ of the positive drug IMA for K562 cells was 0.20 ± 0.02 μM (**Figure 1B**), and the inhibition effect of IMA on the other cell lines was weaker than the K562 cell line. According to the dose-effect curve of HJB and IMA, the concentrations of HJB used in the following study were 3.60, 14.44, and 57.80 μM, and the concentrations of IMA used were 0.05, 0.20, and 0.50 μM. Besides, the study of HJB on a healthy macrophage RAW 264.7 cell line showed relative low toxicity, i.e., the inhibition rate was 39.4% ± 1.6% when the concentration of HJB was 101.8 μM (Supplementary Figure S5).

### Effect of HJB on Apoptosis in K562 Cells

**FIGURE 2:**
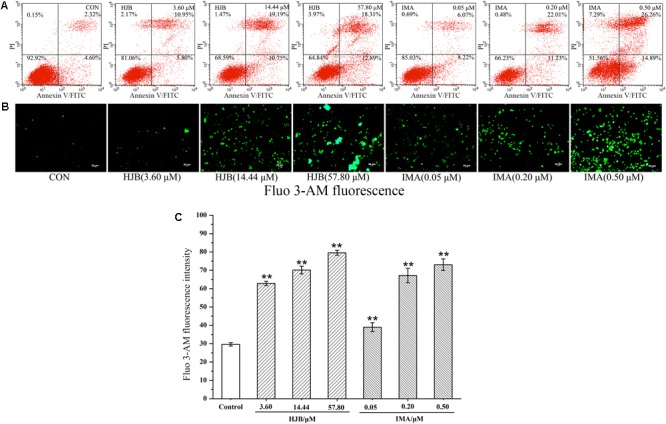
Effect of HJB on apoptosis in K562 cells by flow cytometry and fluorescence microscope. **(A)** Apoptosis was analyzed by annexin V/PI staining and flow cytometry of K562 cells treated with the indicated concentrations of HJB and IMA for 48 h. Representative FACS scatter-grams are shown. **(B)** Fluorescence images of HJB and IMA on Ca^2+^ homeostasis in K562 cells. **(C)** Fluorescence intensity measured by the image analyzer. K562 cells were treated with different concentrations of HJB and IMA for 24 h. Data represented the mean ± SD of three independent experiments, where each sample was tested in triplicate. Representative fluorescence images were taken at 200×. ^∗^*p* < 0.05, ^∗∗^*p* < 0.01 vs. control group.

The apoptosis of K562 cells induced by HJB was investigated using the Annexin V-FITC Kit followed by flow cytometric analysis, and we found a dose-dependent apoptotic effect of HJB in K562 cells (**Figure 2A**). The apoptotic cells (Annexin V positive/PI negative) were 4.6% for the control group. After treatment with different concentrations of HJB (3.60, 14.44, and 57.80 μM), the cell apoptosis rates increased to 5.80, 10.75, and 12.89%, respectively. In addition, while the cell rate with both apoptotic and necrotic markers (Annexin V positive/PI positive) was 2.32%, it increased to 10.95, 19.19 and 18.13%, respectively, with increasing HJB concentrations (3.60, 14.44, and 57.80 μM). The results also show that the cell rate in the third quadrant was 6.07, 22.01, and 26.26%, respectively, with increasing IMA concentration (0.05, 0.20, and 0.50 μM).

### Effect of HJB on Δψm in K562 Cells

**FIGURE 3:**
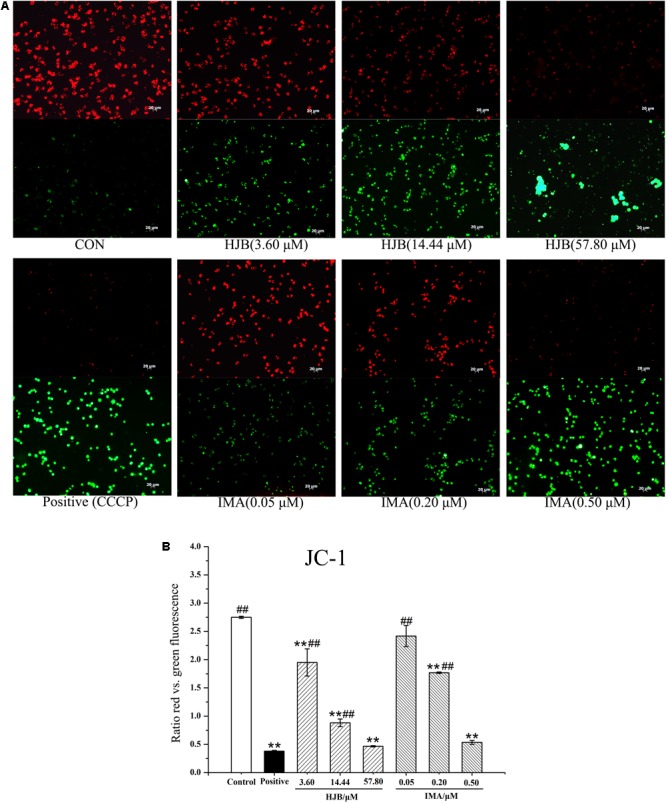
Effect of HJB and IMA on mitochondrial membrane potential in K562 cells. **(A)** Fluorescence images of JC-1-stained K562 cells treated with HJB. **(B)** Relative fluorescence intensity (the ratio red vs. green fluorescence) measured by the image analyzer. K562 cells were treated with different concentrations of HJB and IMA for 24 h. The results are representative of three independent experiments. Representative fluorescence images were taken at 200×. ^∗^*p* < 0.05, ^∗∗^*p* < 0.01 vs. control group; ^#^*p* < 0.05, ^##^*p* < 0.01 vs. CCCP group.

Normal Δψm is required to maintain oxidative phosphorylation and produce ATP, and is necessary to maintain the function of mitochondria. Decreased Δψm has a significant effect on intracellular energy metabolism and apoptosis. JC-1 is a potential-dependent fluorescent dye that forms a polymer under the conditions of normal membrane potential in the mitochondrial matrix. K562 cells were stained with JC-1 and observed by fluorescence microscopy. Cells in the control group demonstrated strong red fluorescence and weak green fluorescence, indicating that the cells were in good condition (**Figure 3A**). In contrast, with the increase of HJB concentration, the red fluorescence intensity decreased while the green fluorescence increased (**Figure 3B**). The relative fluorescence intensity (red/green) measured by the image analyzer significantly decreased (*p* < 0.01), indicating that the Δψm decreased. These results show that HJB and IMA have a significant influence on the Δψm of K562 cells, and that the apoptosis induced by HJB is possibly related to this change in Δψm.

### Effect of HJB on Ca^2+^ Homeostasis and Expression Level of TRPC6 in K562 Cells

**FIGURE 4:**
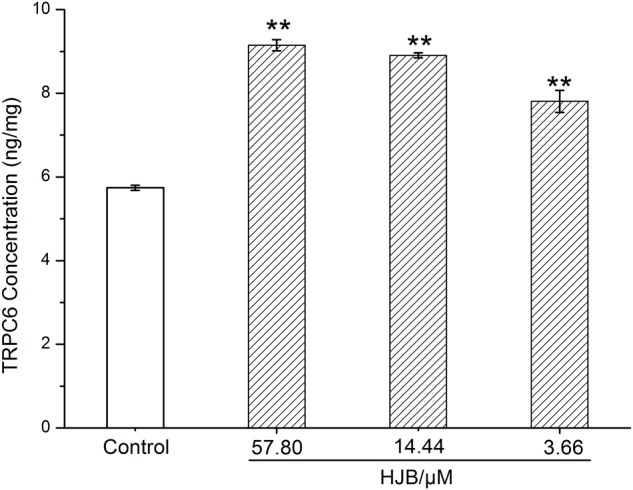
Surface expression level of TRPC6 in K562 cells treated with different concentrations of HJB for 24 h. The quantification was performed using ELISA, and the results are representative of three independent experiments. ^∗^*p* < 0.05, ^∗∗^*p* < 0.01 vs. control group.

We designed to determine cytosolic Ca^2+^ levels and TRPC6 expression in this section. Ca^2+^ acts as a second messenger playing a key role in signal transduction, and activates a variety of intracellular enzymes. When cells are damaged, Ca^2+^ influx is increased, and intracellular Ca^2+^ homeostasis is destroyed, which leads to the destruction of important macromolecules that sustain cell structure and function. We used the Ca^2+^ probe Fluo 3-AM to display cytosolic Ca^2+^ levels by fluorescence microscopy. While the fluorescence of the Ca^2+^ in the control group was weak, treatment with different concentrations of HJB or IMA obviously increased intracellular Ca^2+^ fluorescence (*p* < 0.01), indicating that cytosolic Ca^2+^ concentration increased (**Figures 2B,C**). As the dose increased, the concentration of Ca^2+^ increased accordingly, showing a dose-dependent effect. Besides, the cytotoxicity and Ca^2+^ homeostasis assays of HJB were also performed in Ca^2+^ free media, and the results showed that the IC_50_ of HJB was 15.96 μM, demonstrating little difference with the previous assay that the IC_50_ was 15.07 ± 1.01 μM (Supplementary Figure S3). In addition, under the same concentrations, HJB could also obviously increase Ca^2+^ fluorescence compared with the control group (*p* < 0.01) (Supplementary Figure S4). The results support that HJB increased cytosolic Ca^2+^ level by increasing release of Ca^2+^ from intracellular Ca^2+^ stores, such as the ER of mitochondria. In order to provide further pharmacological evidence for aberrant calcium caused by HJB, we designed to analyze TRPC6 expression which plays an important role in the regulation of calcium homeostasis in cell signaling. The result indicated that, after treatment of HJB, the level of TRPC6 in K562 cells significantly increased (**Figure 4**). The experiment indicates that HJB influences the expression level of TRPC6, which may affect the release of Ca^2+^ from mitochondria.

### HJB Enhances Caspase-8 and Caspase-9 Levels in K562 Cells

**FIGURE 5:**
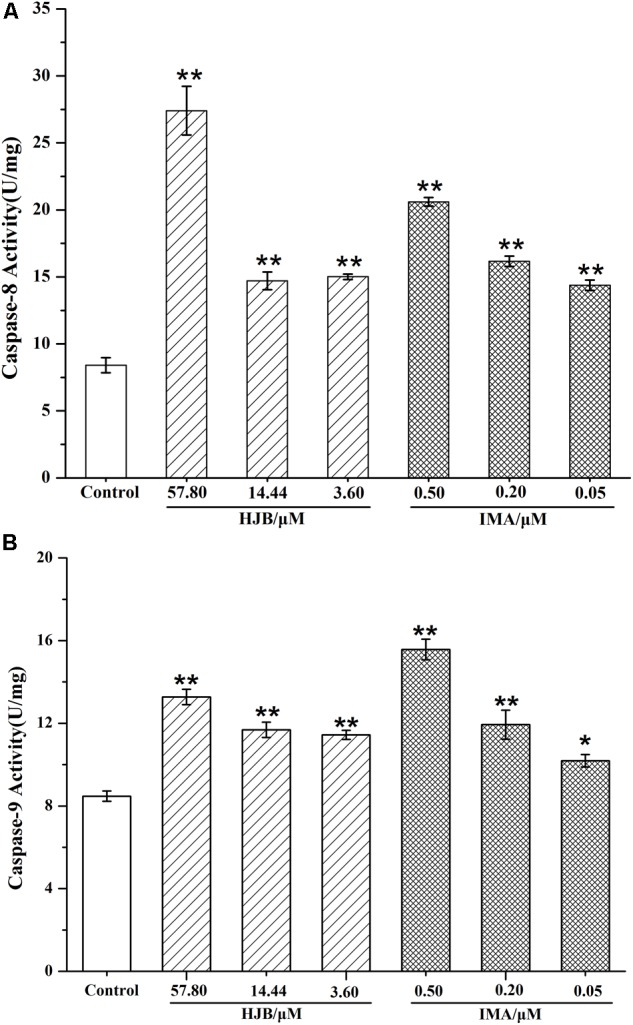
Caspase-8 **(A)** and caspase-9 **(B)** activities in K562 cells after treatment by HJB and IMA. The quantification was performed using ELISA. Values were expressed as the mean ± SD. ^∗^*p* < 0.05, ^∗∗^*p* < 0.01 vs. control group. All results are representative of three independent experiments.

To confirm the action of HJB in apoptosis, we detected the expression of pro-apoptotic proteins, caspase-8 and caspase-9. The activity of caspases in K562 cells treated with HJB and IMA was calculated by the amount of caspase-8 and caspase-9 that cleaved 1.0 nmol of the colorimetric substrate Ac-IETD-*p*NA/Ac-LEHD- *p*NA per hour at 37°C. Compared with the control group, the activity of caspase-8 and caspase-9 was significantly increased after treatment with different concentrations of HJB (*p* < 0.01) (**Figure 5**). After treatment with different concentrations of the positive drug IMA, the activity of caspase-8 and caspase-9 showed a dose-dependent effect, with activity significantly higher than the control group. These results suggest that HJB-induced apoptosis involves a caspase-dependent pathway.

### Effects of HJB on the Expression of Cytochrome c

**FIGURE 6:**
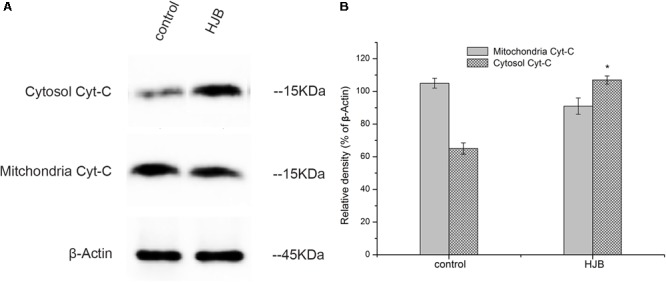
Changes of cytosolic and mitochondrial levels of cytochrome c after treatment with HJB for 24 h. **(A)** Western blotting analysis. **(B)** Results of optical density analyses of cytochrome c/β-Actin. The results are representative of three independent experiments. ^∗^*p* < 0.05 vs. control group.

Upon apoptotic stimulation, cytochrome c released from mitochondria prime a caspase-9-activating complex in the cytosol. An obvious increase in the amount of cytochrome c in the cytosol was detected after treatment with 57.8 μM HJB for 24 h, gray ratio analysis revealed that cytochrome c expression was obviously up-regulated compared with control (*p* < 0.05). However, in this study, the mitochondrial level of cytochrome c after treatment with HJB had no significant variation compared with control (although a down-regulated trend was shown) (**Figure 6**). Therefore, further study is needed to provide evidence for the release of cytochrome c from mitochondria, taking different time points treated by HJB into account.

### HJB Enhances p53 Levels in K562 Cells

**FIGURE 7:**
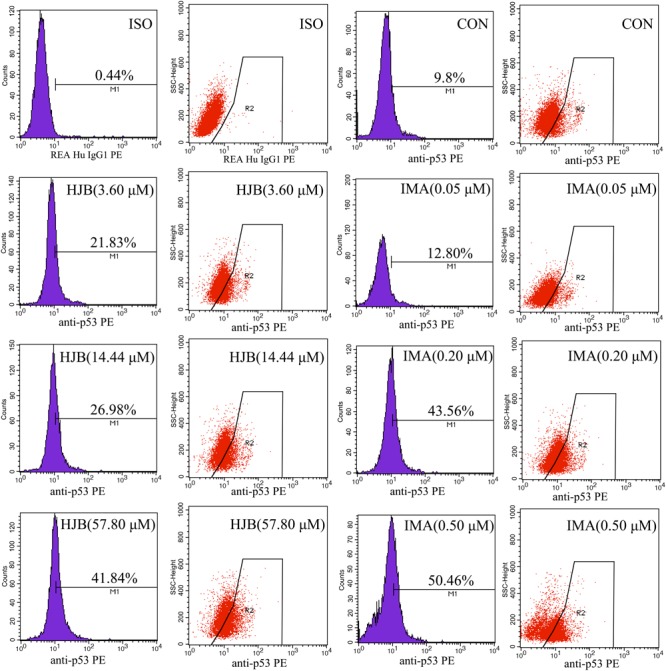
Effect of HJB and IMA on the expression of p53 in K562 cells. K562 cells were treated with different concentrations of HJB and IMA for 24 h, and intracellular p53 was labeled with Anti-p53 pS37-PE and analyzed by flow cytometry. The results are representative of three independent experiments.

The expression of p53, a transcription factor that regulates apoptosis and the cell cycle, was investigated in this study. Following treatment with different concentrations of HJB for 24 h, intracellular p53 was labeled with Anti-p53 pS37-PE and analyzed by flow cytometry. The isotype control group was used to subtract non-specific adsorption in cells and to locate the stained cell population. Expression of p53 increased in a dose-dependent manner (**Figure 7**). While only 9.8% of the control group expressed p53, it increased to 21.83, 26.98, and 41.84% after treatment with increasing concentrations of HJB. Similarly, after IMA treatment, p53 expression increased to 12.80, 43.56, and 50.46%. These results confirm that HJB-induced apoptosis is correlated with the expression of intracellular p53.

### Pharmacokinetics in Rats

We developed and validated an accurate and sensitive UFLC-ESI-MS/MS method to determine HJB levels in rat plasma samples. The chromatographic methods, mass spectrometry conditions, and validation parameters met the analysis requirements of HJB described by the ICH guidelines (Supplementary Materials: Text 13 and Results: Text 14–18). The [M+H]^+^ ions and chromatograms of HJB are shown in Supplementary Figures S1, S2. The extraction recovery and matrix effect results are summarized in Supplementary Table S1.

**FIGURE 8:**
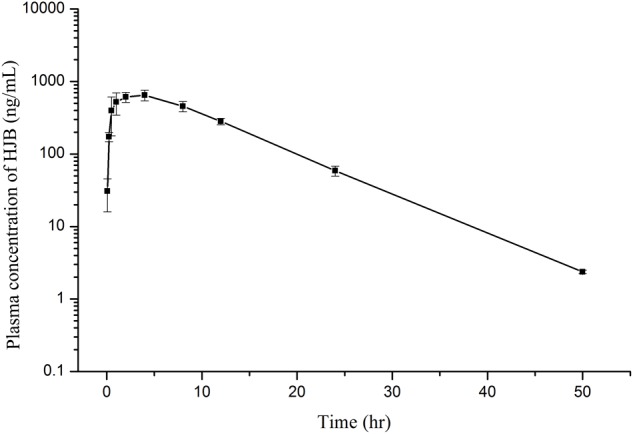
Mean plasma concentration–time profiles of HJB determined by LC-ESI MS/MS method after oral administration of 1.20 mg/kg HJB to rats. Each point represents mean ± SD (*n* = 3).

The plasma concentration-time profiles of HJB in rats are shown in **Figure 8** and the main pharmacokinetic parameters of HJB after oral administration are presented in **Table 1**. After oral administration at the dose of 1.20 mg/kg, HJB was rapidly absorbed, reaching a mean *C*_max_ of 680.7 ± 102.6 ng/mL at a *T*_max_ of 2.67 ± 1.33 h. The elimination half-life (*t*_1/2_) value was estimated to be 5.53 ± 0.15 h. The mean area under the plasma concentration–time curve from time zero to the last measurable plasma concentration point (AUC_last_) was 8676.1 ± 873.0 (h × ng)/mL and the mean area under the plasma concentration–time curve from time zero to time infinity (AUC_Inf_) was 8695.1 ± 871.8 (h × ng)/mL. Clearance (CL), mean residence time from time zero to time infinity (*MRT*_Inf_), and volume of distribution (*V*_z_/*F*) values were estimated to be 0.14 ± 0.01 L/h/kg, 9.36 ± 0.29 h, and 1.11 ± 0.15 L/kg, respectively.

**Table 1:** Main pharmacokinetic parameters of HJB in rats determined after oral administration at 1.20 mg/kg HJB (*n* = 3, mean ± SD).

PK parameters	Unit	HJB
*C*_max_	ng/mL	680.7 ± 102.6
*T*_max_	h	2.67 ± 1.33
*t*_1/2_ Lambda_z	h	5.53 ± 0.15
AUC_last_	h × ng/mL	8676.1 ± 873.0
AUC_Inf_	h × ng/mL	8695.1 ± 871.8
AUC_Extr_	%	0.22 ± 0.04
*V*z/F	L/kg	1.11 ± 0.15
Cl/F	L/h/kg	0.14 ± 0.01
MRT_last_	h	9.25 ± 0.28
MRT_Inf_	h	9.36 ± 0.29

## Discussion

K562 is a hematopoietic progenitor cell line established from a human CML patient in blast crisis (Lozzio and Lozzio, 1975), and it is considered as a classical CML cell model because of its BCR-ABL positive phenotype (Wang et al., 2016). This cell line is non-adherent and unable to proceed with differentiation (Yang et al., 2016). Since CML cells are highly resistant to chemotherapeutic drugs (O’Hare et al., 2006), the exploration of new therapeutic agents and their molecular mechanisms is important for improving the outcomes of patients with human CML (Yang et al., 2016).

6′-hydroxy justicidin B, an arylnaphthalide lignan, has a similar structure to HJA, HJC, and JB, all of which were isolated from *J. procumbens* (Yang et al., 2006; Hemmati and Seradj, 2016). In previous studies, *J. procumbens* lignans showed favorable antitumor qualities (Lee et al., 2005; Won et al., 2015). In the present study, HJB also showed a stronger effect on K562 and HL60 cell lines than on the HepG2, MDA-MB-157, and MGC-803 cell lines. The IC_50_ of HJB on K562 cells was similar to that of HJC, but it was significantly lower than what has been observed for HJA, JB, CME, and TEME (Luo et al., 2014a). These results suggest that HJB is a promising and worth-while antitumor agent, especially for the therapy of hematological malignancy.

In our cell pharmacological studies, HJB induced apoptosis in K562 cells. Increasing HJB concentration enhanced cytotoxicity in K562 and increased both early and late apoptosis in a dose-dependent manner and to a greater extent than HJC. IMA is a tyrosinase inhibitor that blocks one or more protein kinases. It is used for the treatment of chronic myeloid leukemia and malignant gastrointestinal stromal tumors in clinical trials. IMA, as a positive drug, showed similar trends as HJB on K562 cells, with a significantly stronger effect than POD and ETO (Luo et al., 2014b).

Mitochondria maintain normal levels of oxidative phosphorylation, which is involved in regulation of cell replication, growth, and differentiation processes (Moncada and Erusalimsky, 2002; Inoue et al., 2003; Cadenas, 2004). The permeability of the mitochondrial membranes is different. Carriers and channels on the inner membrane, such as proton pumps, form the Δψm by impelling protons out of the mitochondrial matrix. The Δψm is necessary for mitochondrial function (Kroemer et al., 2001). The role of mitochondria in cell apoptosis has aroused plenty of attention in recent years. In the mitochondrial apoptotic pathway, excessive opening of mitochondrial permeability transition pores leads to a decrease of Δψm disrupting the homeostasis of Ca^2+^ in the mitochondrial matrix. This causes several pro-apoptotic factors to be released such as cytochrome c and apoptosis induced factor (AIF) and leads to activation of the caspase family (Marchetti et al., 1996; Korge and Weiss, 1999; Cheng et al., 2006; Leytin et al., 2009). In this study, the Δψm of K562 cells was decreased after 24 h of treatment with HJB, and the effect was enhanced when the dose was increased. In previous studies, HJA and HJC have been reported to cause changes in intracellular redox levels (Luo et al., 2014b). This suggests that HJB, JA and HJC disrupt redox levels and the electron transport chain leading to cell apoptosis.

The endoplasmic reticulum (ER) is the main site of intracellular protein synthesis, folding, and post-translational modification. It is also major storage site of Ca^2+^ that is used for Ca^2+^ signal transduction. Ca^2+^ is a second messenger for multiple death signaling pathways and has a complex relationship with mitochondrial function and ROS (Malhotra and Kaufman, 2011; Bhandary et al., 2012). Upon stimulation, the ER releases its stored Ca^2+^. The increase of mitochondrial Ca^2+^ influx may lead to mitochondrial damage causing an increase of ROS as well as the release of cytochrome C, which induces apoptosis (Kadenbach et al., 2004). ROS cause serious damage to multiple organelles in the cell, which also accelerates apoptosis. Using a Ca^2+^ probe (Fluo 3-AM), we show that HJB induces an increase in cytosolic Ca^2+^ concentration in a dose-dependent manner (**Figure 1B**). In combination with previous studies, these results suggest that HJB-induced apoptosis is related to loss of cell redox levels and ER stress.

The cysteinyl aspartate-specific proteinase (Caspase) family mediates programmed death in mammalian cells. According to previous studies, HJA has an effect on the activity of caspase-3, -8, and -9 and subsequent apoptosis depends on activation of the caspase pathway (He et al., 2012). In this study we show that HJB, which has a similar structure to HJA, can increase the activity of caspase-8 and caspase-9 in K562 cells. We conclude that the HJB-induced apoptosis involves a caspase-dependent pathway.

The p53 gene has attracted extensive attention in cancer treatment and pathology because of its high mutation rate and key role in tumor formation. Typically, the p53 gene functions in cell replication, senescence, apoptosis, and DNA repair (Duffy et al., 2017). Recent studies have shown that p53 regulates cancer through adjusting cellular metabolism and ROS levels (Bieging et al., 2014; Kruiswijk et al., 2015). It has been reported that some apoptosis-stimulating factors induce the rapid transfer of p53 to the mitochondrial membrane, which contributes to apoptosis by directly affecting the structure and function of mitochondria (Matoba et al., 2006; Moll et al., 2006). As a transcription factor, p53 regulates cell apoptosis by up-regulating apoptotic genes and down-regulating anti-apoptotic genes (Zheng, 2017). Using Anti-p53 pS37-PE to label the p53 protein we found that p53 levels were significantly increased after HJB treatment, indicating that HJB-induced apoptosis of K562 cells might utilize the p53 pathway.

In order to verify the ability of HJB in human leukemia therapies, the effect of HJB on the HL60 cell line that represent a human promyelocytic leukemia cells BCR/ABL negative cells (acute myeloid leukemia) was investigated. The methods for cytotoxicity, Δψm and Ca^2+^ homeostasis assay were in line with K562 assays. The results showed that HJB had strong cytotoxicity on HL60 cell line, with the IC_50_ of 1.63 μM (Supplementary Figure S5). The activity of caspase-8 in HL60 cells was obviously increased after treatment with 6.36 μM HJB (*p* < 0.01) (Supplementary Figure S6). Besides, with the increase of HJB concentration, the Δψm decreased significantly (*p* < 0.01) (Supplementary Figure S7). In addition, it was demonstrated that, after treatment of HJB, cytosolic Ca^2+^ concentration increased and Ca^2+^ homeostasis was destroyed (Supplementary Figure S8), showing a dose-dependent effect. Furthermore, after treatment of HJB, the level of SERCA in HL60 cell supernatants significantly decreased while the level of TRPC6 in cell extracts significantly increased (Supplementary Figure S9), demonstrating that HJB might regulate the level of SERCA and TRPC6 to affect Ca^2+^ homeostasis that induced apoptosis of HL60 cells.

Additionally, we performed a study on the pharmacokinetics of HJB *in vivo*. Compared with previous studies (Qiu et al., 2012, 2013; Zhou et al., 2012; Luo et al., 2017), the elimination *t*_1/2_ and MRT_last_ values of HJB were significantly larger than those of HJA, TEME, JB, and CME. In contrast, HJB’s CL value is significantly lower. The *C*_max_ of HJB is 680.7 ± 102.6 ng/mL, which is higher than the *C*_max_ of HJA, TEME, JB, and CME. In addition, the rate of absorption of HJB is rapid when the blood concentration is lower, so that HJB has a longer residence time *in vivo*.

In conclusion, this study mainly demonstrated strong cytotoxic activity of HJB on K562 cells, and HJB triggers a critical imbalance in Ca^2+^ homeostasis and mitochondrion-dependent cell death. In addition, HJB showed good pharmacokinetic characteristics, which makes it a promising drug for the treatment of human leukemia. However, *in vivo* activity and toxicity of HJB remain to be elucidated in the future research.

## Conclusion

In this study, the cytotoxic activity of HJB, an arylnaphthalene lignan isolated from *J. procumbens*, on K562 cells was studied systematically. The results indicate that HJB induces apoptosis in K562 by disrupting Δψm and intracellular Ca^2+^ homeostasis to active the associated signal pathway. HJB treatment significantly increases the activity of caspase-8, caspase-9 and the expression of p53. Our analyses indicate that HJB promotes apoptosis by activating the caspase-dependent pathway and interfering with mitochondria. The study demonstrated the effect of HJB on K562 cells and its potential for the treatment of human leukemia.

Furthermore, an accurate and sensitive UFLC-ESI-MS/MS method with simple liquid-liquid extraction procedures was developed and validated for studying the pharmacokinetics of HJB in rat plasma samples. The elimination half-life (*t*_1/2_), mean residence time (MRT), and clearance (CL) of HJB were estimated to be 5.53 ± 0.15 h, 9.36 ± 0.29 h, and 0.14 ± 0.01 L/h/kg, respectively. Fast drug metabolism (with quick absorption and fast elimination) might limit administration methods, e.g., frequent intravenous injection. We show that HJB has good absorption properties and moderate metabolism *in vivo*, which are favorable characteristics for antineoplastic agents.

## Author Contributions

JL and MY designed the study. JL and JQ performed the experiments. JL, YF, and SZ analyzed the data. JL and JQ wrote the paper. XZ and MY revised the paper.

## Conflict of Interest Statement

The authors declare that the research was conducted in the absence of any commercial or financial relationships that could be construed as a potential conflict of interest. The reviewer DT and handling Editor declared their shared affiliation.
