# Supplementary material for: 6′-Hydroxy Justicidin B Triggers a Critical Imbalance in Ca2+ Homeostasis and Mitochondrion-Dependent Cell Death in Human Leukemia K562 Cells
Source: Front Pharmacol. 2018 Jun 6;9:601. doi: 10.3389/fphar.2018.00601 (PMC6008565; doi:10.3389/fphar.2018.00601)
Supplement: Supplementary file 1 [file Data_Sheet_1.doc]

**Supplementary** **Materials**

**1. Text files**

***Text 1:*** ***Materials***

Dimethyl sulfoxide (DMSO) was purchased from sigma (St. Louis, MO, USA). The fetal calf serum, RPMI medium 1640 (1×), 0.25% trypsin, 3-(4,5-Dimethylthiazol-2-yl)-2,5 -diphenyltetrazolium bromide, phosphate buffer salin e (PBS) and penicillin-streptomycin solution (100×) were purchased from Corning CellGro® (Herndon, Virginia, USA). Annexin V-FITC Kit including Annexin V-FITC, 20×Binding Buffer Stock Solution and Propidium Iodide (PI), Anti-p53 pS37-PE, human and REA Control (I) antibodies were purchased from Miltenyi Biotec (München, Germany). Foxp3 Staining Buffer Set was purchased from eBioscience (Washington, D.C., USA) including fixation diluent, fixation reagent and permeabilization buffer. Caspase-8 activity assay kit (Lysis Buffer, Detection buffer, Ac-IETD-*p*NA and *p*NA), Fluo-3 AM, JC-1 Mitochondrial membrane potential assay kit (JC-1 (200×), Ultra-pure water, JC-1 staining buffer (5×) and CCCP (10 mM)) and Bradford protein assay kit (G250 staining solution and 5 mg/mL Bovine Serum Albumin) were purchased from Beyotime Biotechnology (Shanghai, China). HPLC grade formic acid and methanol were obtained from Fisher Scientific Co., Ltd. (Emerson, IA, USA). Ultrapure water was produced by a Milli-Q Water System (Millipore, MA, USA). All other chemicals were of analytical grade.

**2. Supplementary Methods**

***Text2: The chromatographic conditions***

The gradient elution by a mobile phase consisting of water (0.5% formic acid, 10mM NH_4_COOH) (A) and methanol (B) with following gradient: 0.01–2.00 min: linear from 80% A/20% B to 60% A/40% B; 2.00–8.00 min: linear from 60% A/40% B to 50% A/50% B; 8.00–12.00 min: linear from 50% A/50% B to 20% A/80% B; 12.00–12.01 min: switch from 20% A/80% B to 80% A/20% B and hold 80% A/20% B until 18.00 min. The injection volume was 5 μL.

***Text 3: The mass spectrometric conditions***

Following optimization of the setting parameters, the instrument was operated in the positive mode with an ion spray voltage of 5.5 kV, curtain gas pressure of 35 psi, nebulizer gas pressure of 50 psi, heater gas pressure of 50 psi and the source temperature set at 550 ^o^C. The curtain, nebulizer, heater and collision gas were all nitrogen. The multiple reaction monitoring (MRM) conditions for HJB were *m/z* 381.2 → 307.1 (quantitative ions, collision energy (CE): 33), *m/z* 381.2 → 279.1 (CE: 30), *m/z* 381.2 → 337.3 (CE: 29), *m/z* 381.2 → 136.9 (CE: 36) and 381.2 → 275.0 (CE: 33) for HJB, respectively. The MRM condition was *m/z* 386.3 → 122.0 for IS. Information dependent acquisition (IDAs) experiment was performed to explore the fragment pattern in the early method development period. By using MRM as survey scan linked to enhance product ion scans (EPI), the fragment pattern of HJB and potential metabolites were explored with CE setting in the range of 20-50 eV. Q3 entry barrier was 8 V, the scan rate was 20000 Da/s for scan range of 50-450 Da, and collision gas was set to high. The data were collected and analyzed by the Analyst Data Acquisition and Processing software (Version 1.6, Applied Biosystems/MDS Sciex, Concord, ON, Canada).

***Text 4: Cell cultures***

The cell lines were cultured in RPMI-1640 medium with L-Glutamine containing 10% fetal bovine serum, 100 U/mL penicillin and streptomycin. The cells were cultured in CO_2_ incubator that was obtained from SANYO Electric Co., Ltd. (Osaka, Japan) with the temperature of 37 °C and an atmosphere of 5% CO_2_-95% air. The other cell lines (HepG2, MDA-MB-157 and MGC-803) included a digestion procedure with 0.25% trypsin for 2-3 min.

***Text 5: Cell viability assay***

HJB was dis­solved in DMSO and the final concentration of DMSO was 0.1% (*v/v*). HJB was diluted with the culture medium to final concentrations, which ranged from 0.89 to 115.60 μM. The concentration of IMA was ranged from 0.04 to 4.17 μM. HepG2, K562, MDA-MB-157 and MGC-803 cell lines were seeded into 96-well plates at a concentration of 1.0×10^5^ cells/mL. The zero wells were filled with complete medium and different concentrations of HJB-containing medium without cells. The cells in the control group were treated with 0.1% DMSO. The cells in the experimental groups were treated with different concentrations of HJB-containing medium, and each group contains three repeated wells. After incubation at 37 °C in a 5% carbon dioxide incubator for 48 h, 20 μL of 5 mg/mL MTT solution was added to each well and incubated at 37 °C for 4 h. After adding 90 μL of 10% SDS-5% isopropanol-0.012 M HCl, the cells were incubated at 37 °C overnight. The absorbance was measured at 570 nm by using a microplate reader of multi wavelength (Tecan Group Ltd., Switzerland) to calculate the inhibition rate of the cells at different concentrations of HJB. The inhibition rate was expressed by1-[(the experimental group value-zero wells value) / (control group value-zero wells value)] × 100%. The IC_50_ values were calculated by nonlinear regression analysis of the inhibitory rate and drug concentration.

***Text 6: Annexin V/PI staining and flow cytometry analysis***

The logarithmic K562 cells were collected and adjusted with RPMI1640 complete medium into a cell suspension at a concentration of 1.0×10^5^ cells/mL and the cells were added into a six well plate with 1 mL per well. The cells were then treated with different concentrations (3.60, 14.44, 57.80 μM) of HJB in complete medium for 48 h. After the treatment, the cells were collected and cleaned by the binding buffer (1×). The cell concentration was thereafter adjusted and measured using Annexin V-FITC and PI kit in accordance with the manufacturer’s protocol, and apoptosis was finally analyzed by using flow cytometry (BD Pharmingen). Meanwhile, uncolored control group, Annexin V-FITC group and PI single staining group were prepared for flow cytometry adjustment compensation.

***Text 7: Detection of mitochondrial membrane potential***

The positive samples were obtained by adding 10 μmol/L CCCP to cells and incubated for 20 min, which would eliminate the mitochondrial membrane potential. The experimental groups and control group cells were treated with different concentrations (3.60, 14.44 and 57.80 μM) of HJB for 24 h and approximately 5.0×10^5^ cells were collected and resuspended in 0.5 mL complete medium. JC-1 was stained onto K562 cells using JC-1 mitochondrial membrane potential assay kit in accordance with the manufacturer’s protocol. The smear was prepared and observed on a fluorescence microscope (Carl Zeiss, [Jena](http://www.baidu.com/link?url=FLLu-Hl0wwCNEzqm3Z55ZACVeSb2jjyycwWN40kJp_8Oh-2LDW_ABuB9v-06qZ8n0ePR5wGFRO9cEYar6--24RvCLw0zisaJ8-IhQT2JJUq), Germany) and photos were taken to demonstrate the changes of the mitochondrial membrane potential in the cell after treatment. In the process of the fluorescence observation, the green fluorescence was referenced to the FITC-related setting, while the red fluorescence was referenced to Cy3-related settings.

***Text 8: Ca^2+^ homeostasis assay***

Fluo 3-AM was dissolved into DMSO and diluted with HBSS to 5 μmol/L as Fluo 3-AM working solution containing 0.04% pluronic F-127. The Pluronic F-127 was used to prevent the polymerization of Fluo 3-AM in the working solution and promote it entry into the cells. After treated with different concentrations (3.60, 14.44 and 57.80 μM) of HJB for 24h, about 5.0×10^5^ cells was collected and washed by the hank's balanced salt solution (HBSS) for three times. 100 μL of working solution was added into the washed cells, vortex for 30s, and incubated at 37 °C for 30 min. After incubation, HBSS was used to wash the cells for three times and incubated at 37 °C again for 20 min to ensure Fluo 3-AM de-esterified completely. A smear was made and the stained cells were observed under a fluorescence microscope (Carl Zeiss, [Jena](http://www.baidu.com/link?url=FLLu-Hl0wwCNEzqm3Z55ZACVeSb2jjyycwWN40kJp_8Oh-2LDW_ABuB9v-06qZ8n0ePR5wGFRO9cEYar6--24RvCLw0zisaJ8-IhQT2JJUq), Germany).

***Text 9: Caspase-8 activity assay***

Ac-IETD-*p*NA (acetyl-Ile-Glu-Thr-Asp p-nitroanilide) was catalyzed by Caspase-8 to produce pNA (p-nitroaniline), and the absorbance of pNA was measured at 405 nm. According to the total protein in the test samples and the defined of enzyme activity (one unit is the amount of enzyme that will cleave 1.0 nmol of the colorimetric substrate Ac-IETD-*p*NA per hour at 37 °C under saturated substrate concentrations), and the enzyme activity units of caspase 8 was calculated. The K562 cells, about 5.0×10^5^ cells, were collected and treated by different concentration (3.60, 14.44, 57.80 μM) of HJB for 48h, washed once with pre-cooled PBS, and 50 μL of lysis solution was added to incubate with ice for 15 min. The sample lysate was centrifuged at 10000 rpm for 10 min at 4 °C. The supernatant was stored at -70 °C for enzyme activity assay and 10 μL of supernatant was obtained for total protein quantification. The procedure of the measurment of Caspase-9 was the same with Caspase-8 except for the substrate of Ac-LEHD-*p*NA.

***Text 10: p53 expression assay***

p53 is a transcription factor located in the nucleus. Foxp3 staining buffer set was used for the cells fixation and permeabilization. The logarithmic K562 cells were collected and adjusted to 1.0×10^5^ cells/mL, and added to the 6-well plate with 1mL per well. After treated by different concentrations (3.60, 14.44 and 57.80 μM) of HJB for 24 h, the cells were collected and washed by PBS. The cells were stained according to the instructions of the foxp3 staining buffer and analyzed by flow cytometry. The isotype control group was prepared parallelly to subtract nonspecific adsorption interference.

***Text 11: Preparation of standards and calibration curves***

Briefly, the spiking procedure involved transferring 5 μL aliquot of HJB working standard solutions and 50 μL aliquot of blank rat plasma into 1.1 mL centrifuge tubes. QC (quality control) samples were prepared in a similar manner at low, medium and high levels (2.0, 50, 400 ng/mL). The IS working solutions (10 ng/mL) was prepared by diluting with water. All the stock and working standard solutions were stored at 4 °C prior to use.

***Text 12: Pharmacokinetic study in rats***

The dosing solution with HJB concentration of 0.24 mg/mL was prepared by dissolving appropriate amount of HJB in DMSO: water (5:95, v/v). The actual oral dose of HJB was 1.20 mg/kg, and the dose volume was 5.0 mL/kg. After oral administration, aliquots of 0.20 mL blood samples were collected in heparinized polyethylene tubes at different time intervals postdosing (0.083, 0.25, 0.50, 1.0, 2.0, 4.0, 8.0, 12.0, 24.0, 50.0 h). Heparinized blood was centrifuged at 13,000×*g* at 18 °C for 5 min to obtain plasma, which was stored at −80 °C until analysis.

***Text 13: Quantification method validation***

***1) Selectivity, linearity, accuracy, precision, and recovery***

To evaluate the selectivity, six individual samples of blank rat plasma were analyzed by comparing with the spiked analytes for endogenous interferences. The linearity of the method was determined by analyzing a series of standard plasma samples by least squares linear regression of the peak area ratios of HJB to IS obtained against the corresponding concentration (*x*). The limit of quantification (LOQ) was defined as the lowest concentration on the calibration curve with acceptable precision (% RSD≤ 20%) and accuracy (% RE within ±20%). The precision and accuracy of method were assessed by performing replicate analyses of QC samples spiked with low, medium and high concentrations against calibration standards. Five replicates of QC samples at each concentration level were evaluated on the same day for intra-day precision, while repeated analysis at each concentration of QC samples five times per day over five consecutive days for inter-day precision and accuracy. Recoveries of HJB from plasma were determined by comparing the responses in plasma carried through the complete preparation procedure to those spiked into the prepared blank rat plasma of the same concentration as those of QC samples, respectively.

***2) Stability***

The stability of HJB in rats was assessed by analyzing QC samples at three QC concentration levels exposed to different time and temperature conditions. The long-term stability was assessed after the QC samples had been stored at −80 °C for 1 month. The freeze-thaw stability was determined after three freeze-thaw cycles (−20 °C to 20 °C) on 3 consecutive days.

***3) Matrix effects***

Matrix effects were assessed by comparing the peak areas ratios of HJB after addition of low (n=3) and high (n=3) concentrations of HJB to (A) mobile phase and (B) the supernatant of extracted blank plasma (Drug/IS). These studies were conducted with three different lots of plasma. The percentage matrix factor was used as a quantitative measure of the matrix effect.

**3. Supplementary Results**

***Text 14: Chromatography***

This study first describes the development of a sensitive and specific UHPLC-ESI-MS/MS assay for the determination of the content of HJB in rat plasma. The [M+H]^+^ ions of HJB and IS are shown in Fig. S1. Mass chromatograms of HJB and IS obtained by extraction of blank rat plasma, blank rat plasma spiked with the HJB and IS, and actual unknown plasma samples obtained in rats after oral administration of HJB (dose 1.20 mg/kg) are shown in Fig. S2. The chromatographic run time for the extracted plasma samples was 18.0 min (containing 6 minutes of post-time). The retention times for HJB and IS was 4.44 and 6.40 min, respectively. The chromatograms show baseline separation of HJB and the internal standard without any interference from endogenous components.

***Text 15: Linearity, sensitivity and detection limit of the assay***

Calibration standards were prepared by spiking 5 μL of the appropriate standard solutions of HJB to 50 μL of blank rat plasma. The peak area (*y*) and concentration of HJB (*x*) was adopted to calculate calibration equation and correlation coefficients. The LOQ of HJB was 0.50 ng/mL. Typical equation for the standard curves of HJB was *y=*20275.86757*x*-39760.67193 (r=0.9999).

***Text 16: Extraction recovery and matrix effect***

The extraction recovery and matrix effect results are summarized in Table S1. The mean recovery of HJB was 91.6%. The data indicated that the recoveries of HJB from rat plasma were concentration-independent in the concentration range evaluated. In addition, the absolute matrix effect values obtained were all above 80%, which indicated that ion suppression or enhancement from tissue homogenate matrix was negligible.

***Text 17: Accuracy, precision and stability of the assay***

Table S1 summarizes the intra- and inter-day precisions and accuracies of HJB at different concentration levels. The stability results indicated that HJB at the three concentrations tested had acceptable stabilities after three cycles of freeze-thaw, at room temperature for 24 h and at −80 °C for 1 month with the % RE values being within ±15%.

***Text 18: Application of the assay method***

The UFLC-ESI-MS/MS method described was used to quantify HJB in rat plasma samples which were orally administered a dose of 1.20 mg/kg HJB. The result indicated that quantification of the parent compound was not affected by the acquisition of qualitative analysis for the identification of HJB. It’s worth noting that this method is unable to detect unexpected metabolites (Li et al., 2005). For this reason, 5 most intensive MRM transitions for HJB were chosen to reduce the possibility of unpredicted fragmentation.

**Supplementary results**


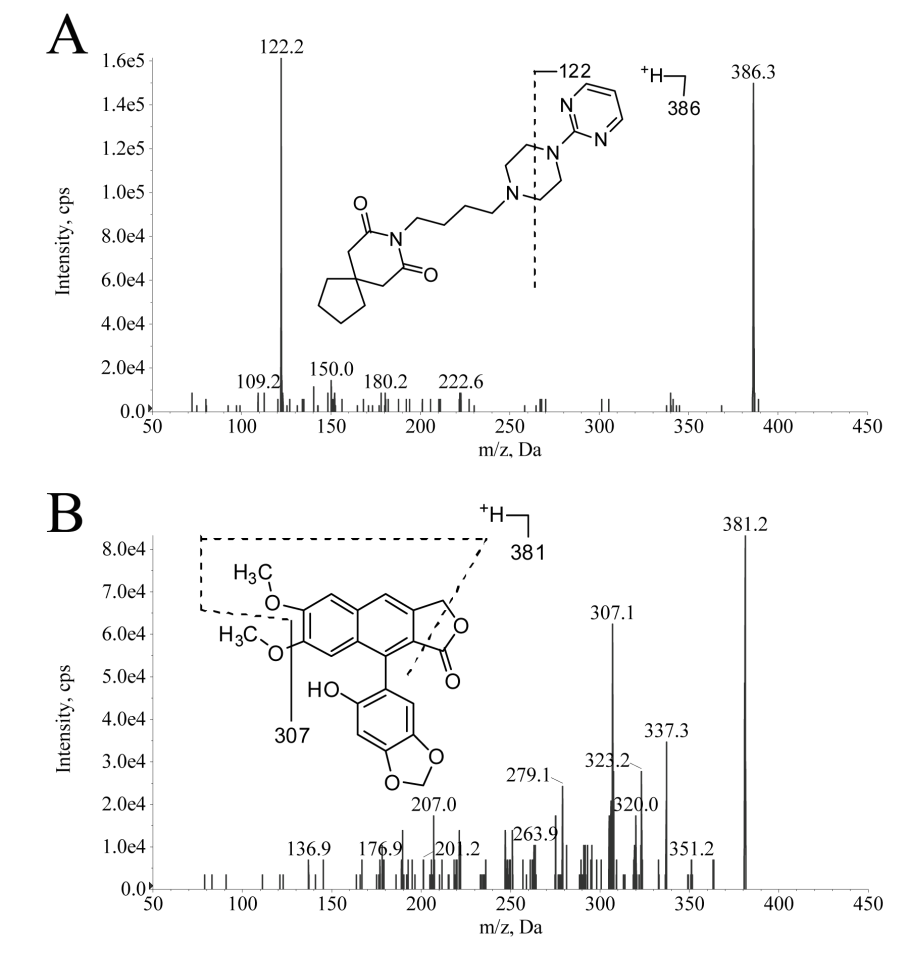


**Figure S1** Full-scan product ion spectra of [M+H]^+^ ions and fragmentation schemes for (A) buspirone (Internal standard) and (B) HJB.


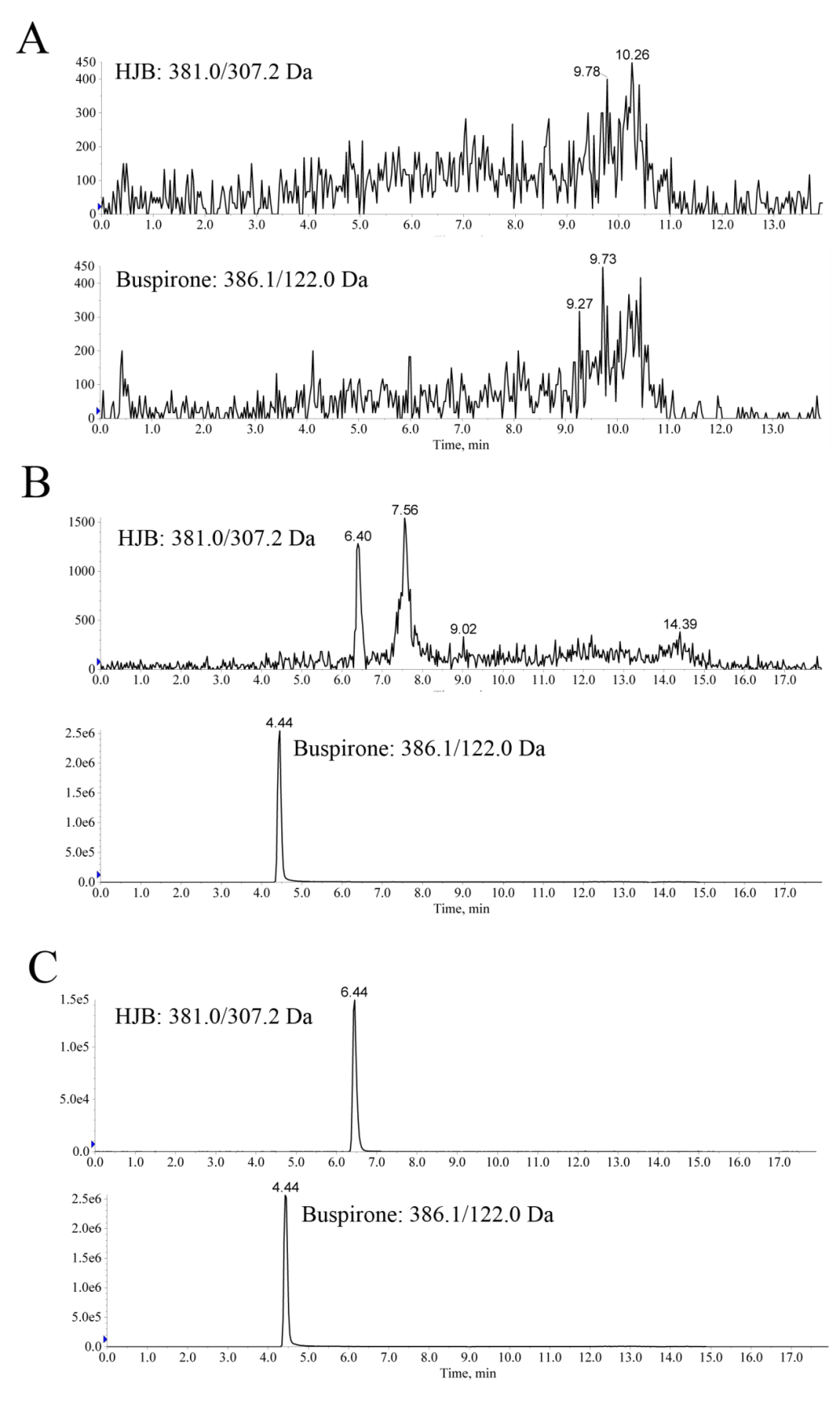


**Figure S2** Typical chromatograms of (A) blank rat plasma; (B) blank rat plasma spiked with HJB (0.50 ng/ml, LOQ) and IS; and (C) an unknown rat plasma sample collected at 24 h after oral administration of 1.20 mg/kg HJB.


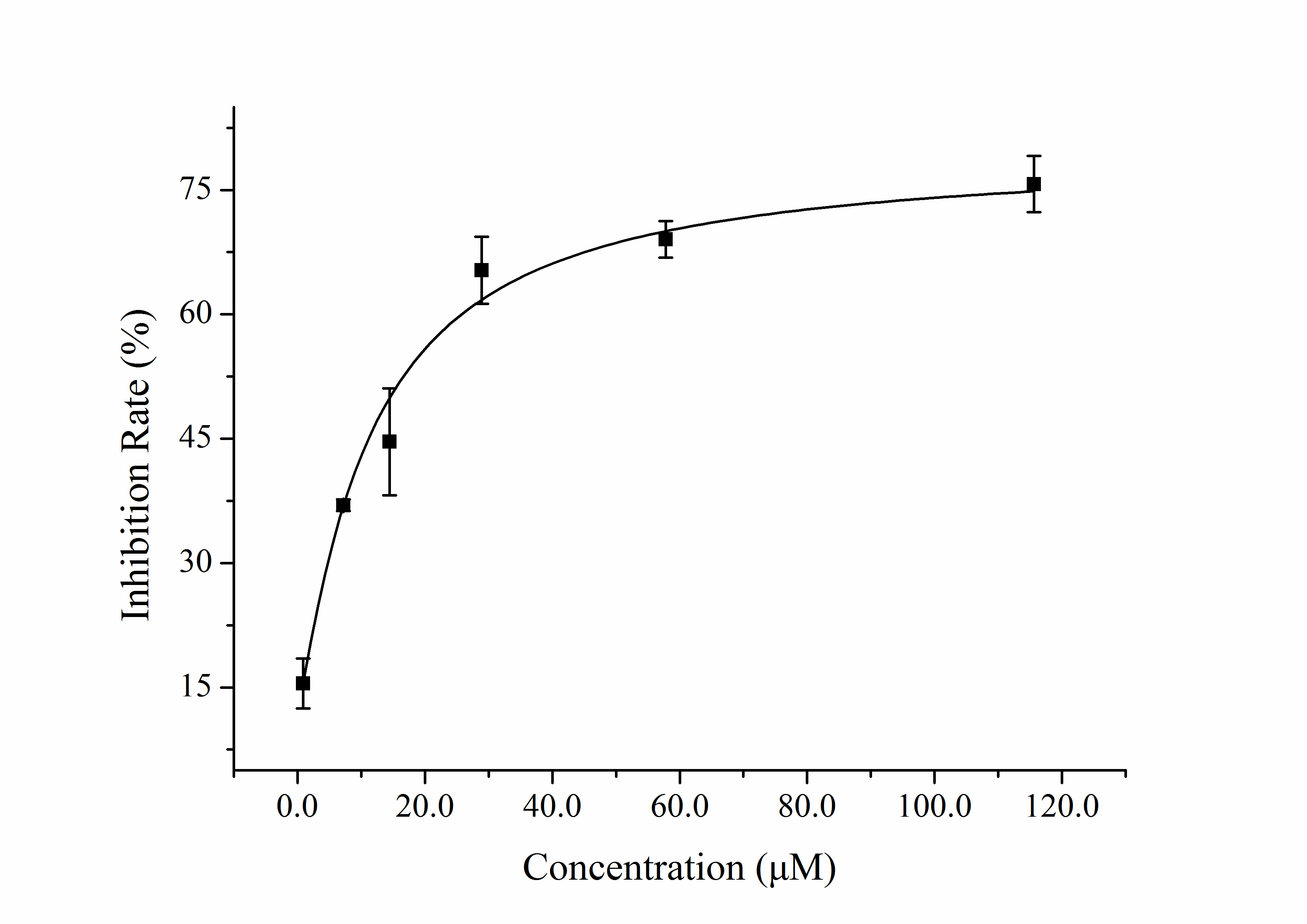


**Figure S3** Cytotoxicity of HJB on K562 cell line in Ca^2+^ free media. The results are representative of three independent experiments.


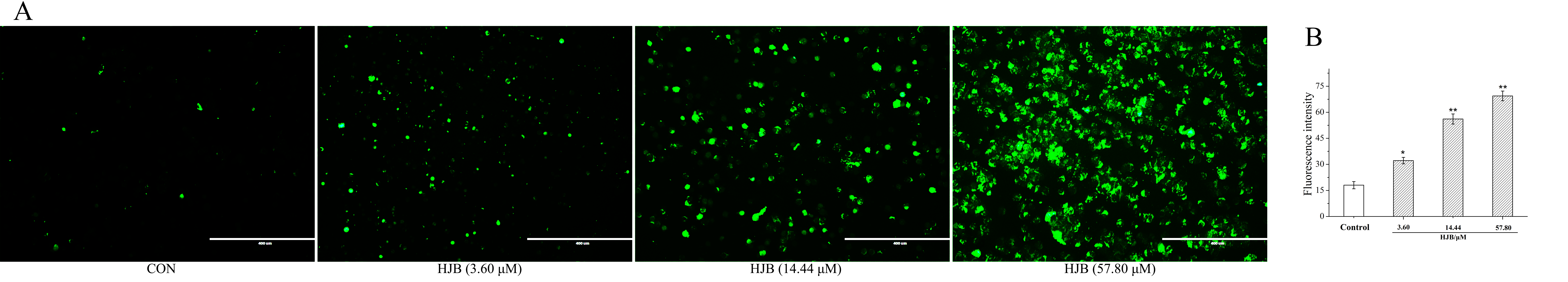


**Figure S4** Effect of HJB on Ca^2+^ homeostasis in K562 cells in Ca^2+^ free media. (A) Fluorescence images of HJB on Ca^2+^ homeostasis in k562 cells; (B) Fluorescence intensity measured by the image analyzer. K562 cells were treated with different concentrations of HJB for 24 h. The results are representative of three independent experiments. Representative fluorescence images were taken at 200×. ^*^*p* < 0.05, ^**^*p* < 0.01 vs. control group.


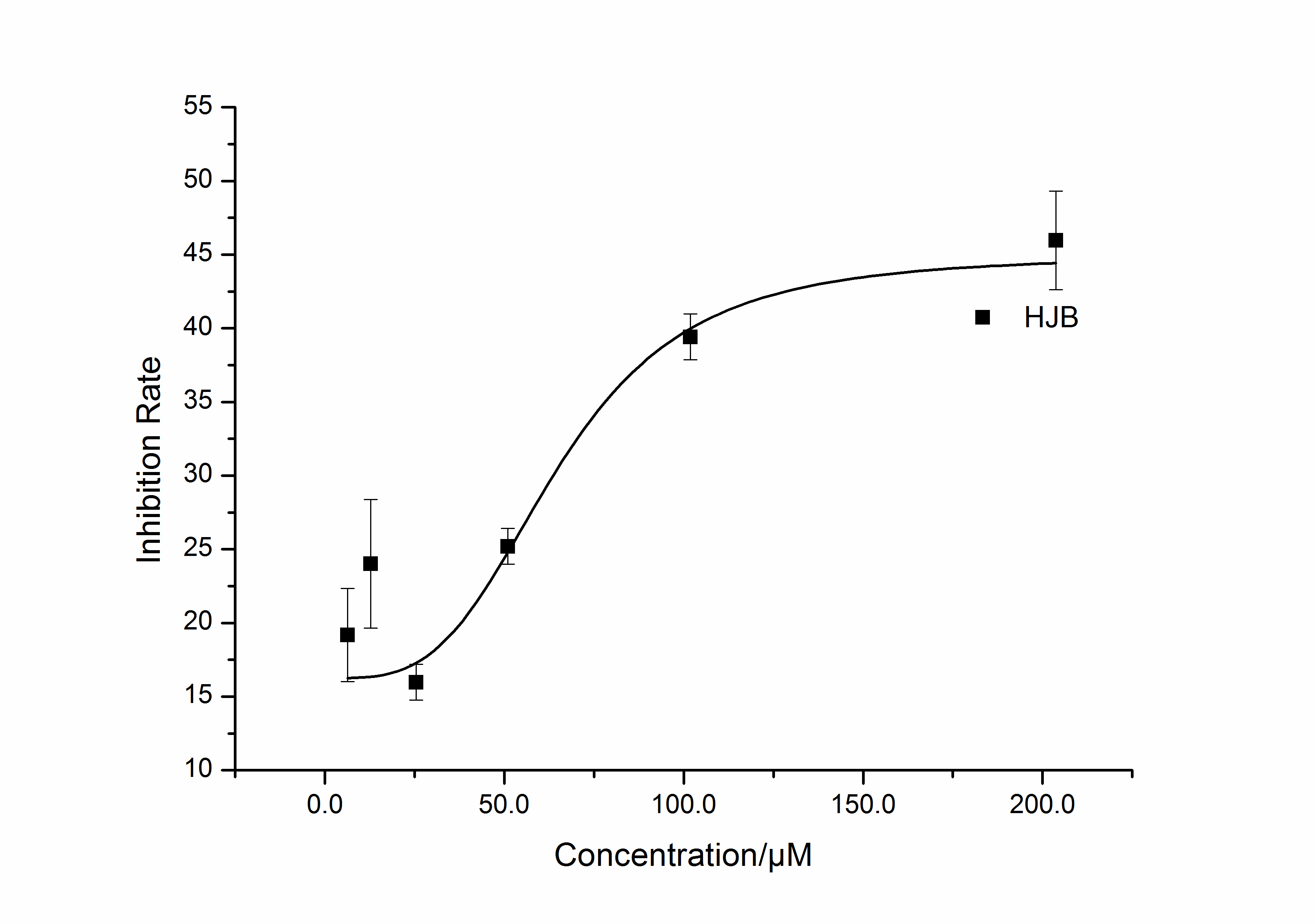

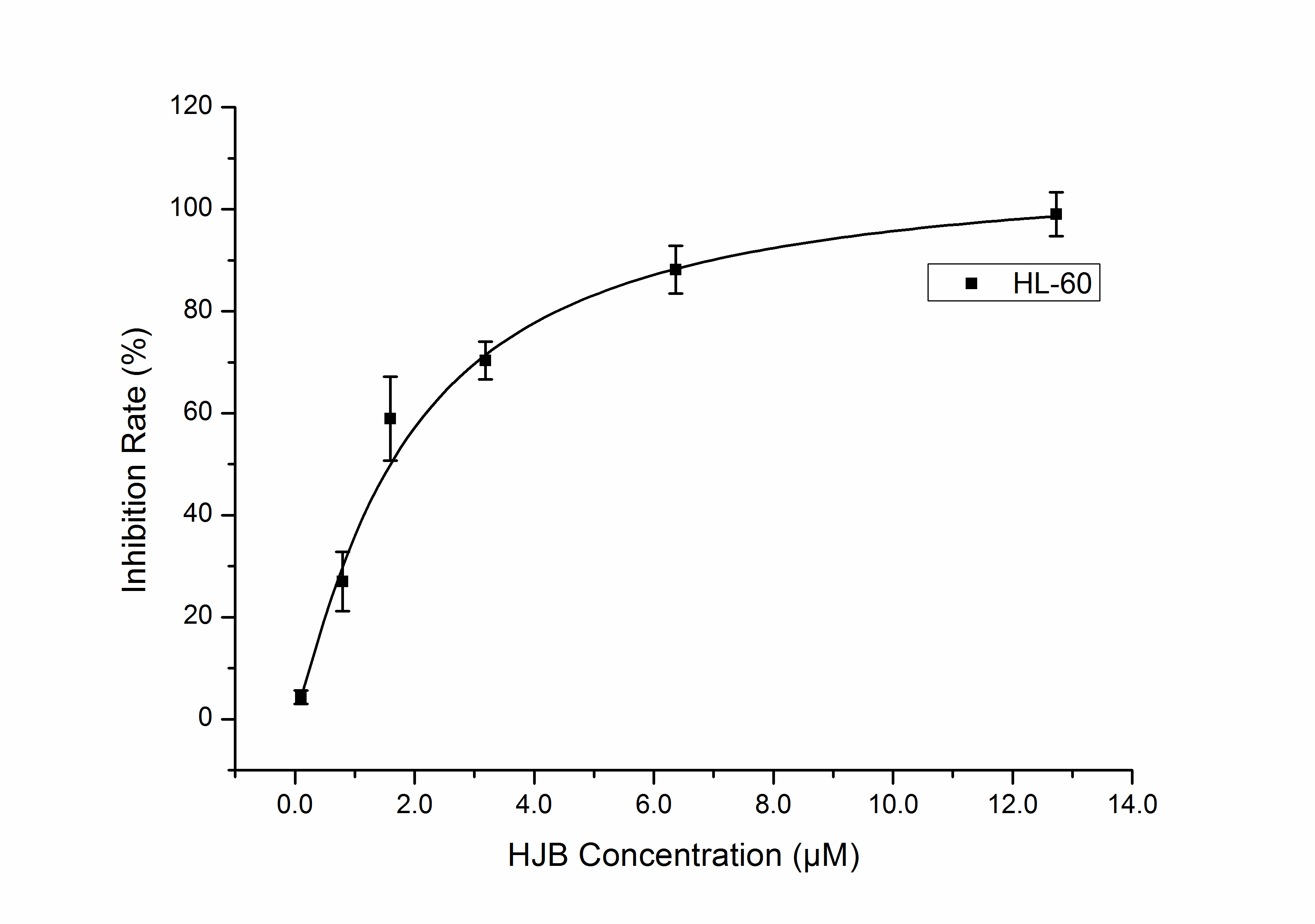


**Figure S5** Cytotoxicity of HJB on HL60 and macrophage RAW 264.7 cell lines. The results are representative of three independent experiments.


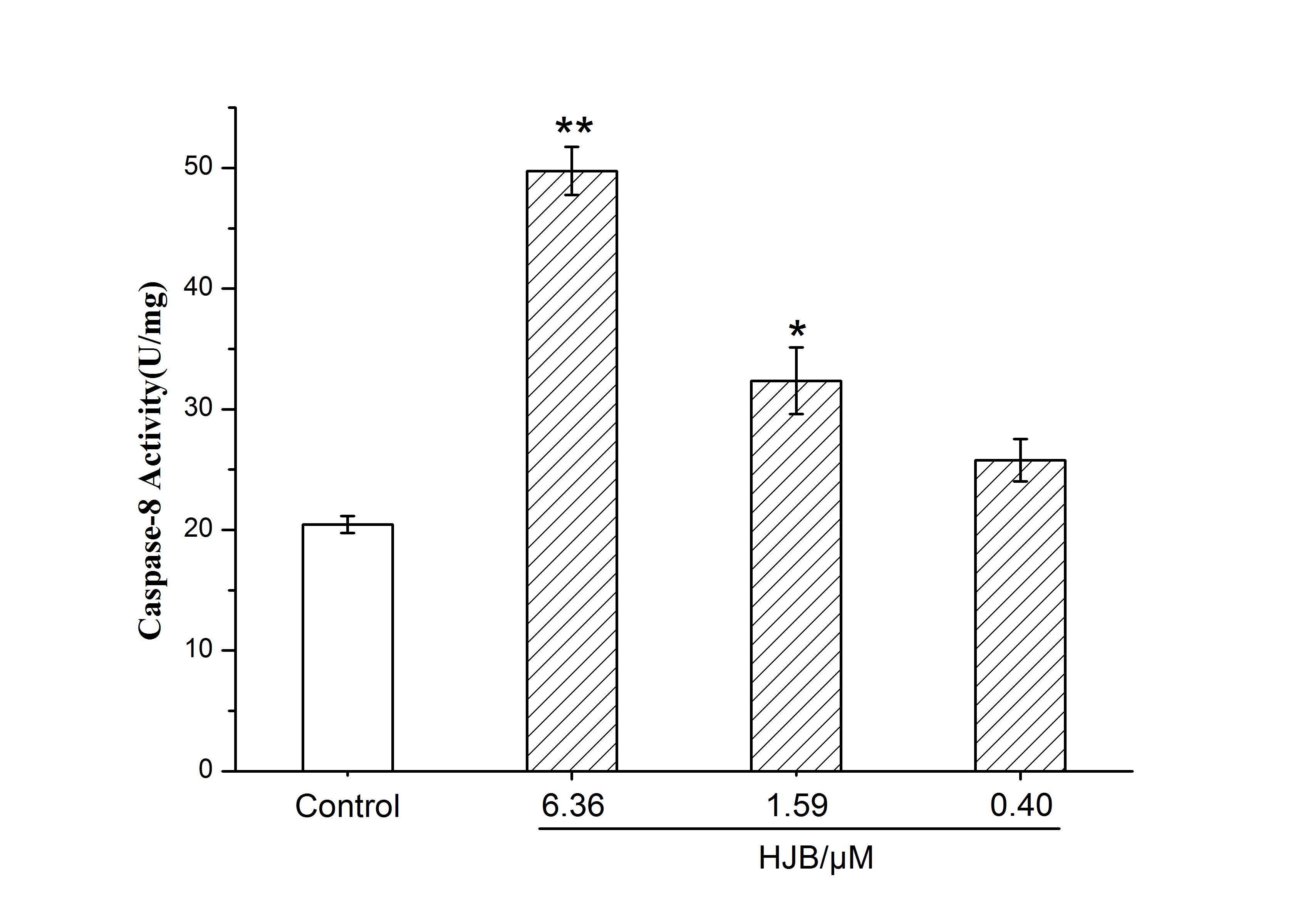


**Figure S6** Caspase-8 activities in HL60 cells after treatment by HJB. Values were expressed as the mean ± S.D. (n=3)





**Figure S7** Effect of HJB on mitochondrial membrane potential in HL60 cells. (A) Fluorescence images of HJB on mitochondrial membrane in HL60 cells; (B) Relative fluorescence intensity measured by the image analyzer. HL60 cells were treated with different concentrations of HJB for 24 h. The results are representative of three independent experiments. Representative fluorescence images were taken at 200×. ^*^*p* < 0.05, ^**^*p* < 0.01 vs. control group; ^#^*p* < 0.05, ^##^*p* < 0.01 vs. CCCP group.


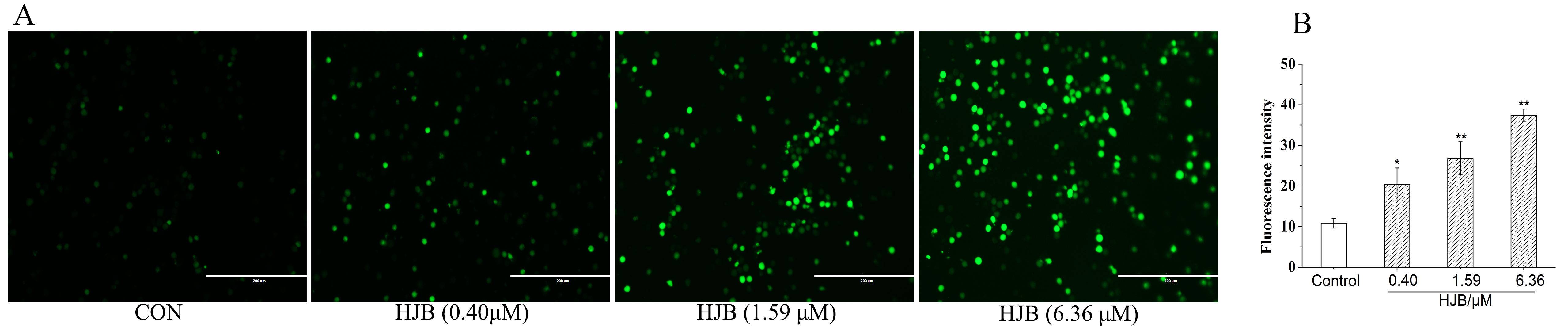


**Figure S8** Effect of HJB on Ca^2+^ homeostasis in HL60 cells. (A) Fluorescence images of HJB on Ca^2+^ homeostasis in HL60 cells; (B) Fluorescence intensity measured by the image analyzer. HL60 cells were treated with different concentrations of HJB for 24 h. The results are representative of three independent experiments. Representative fluorescence images were taken at 200×. ^*^*p* < 0.05, ^**^*p* < 0.01 vs. control group.


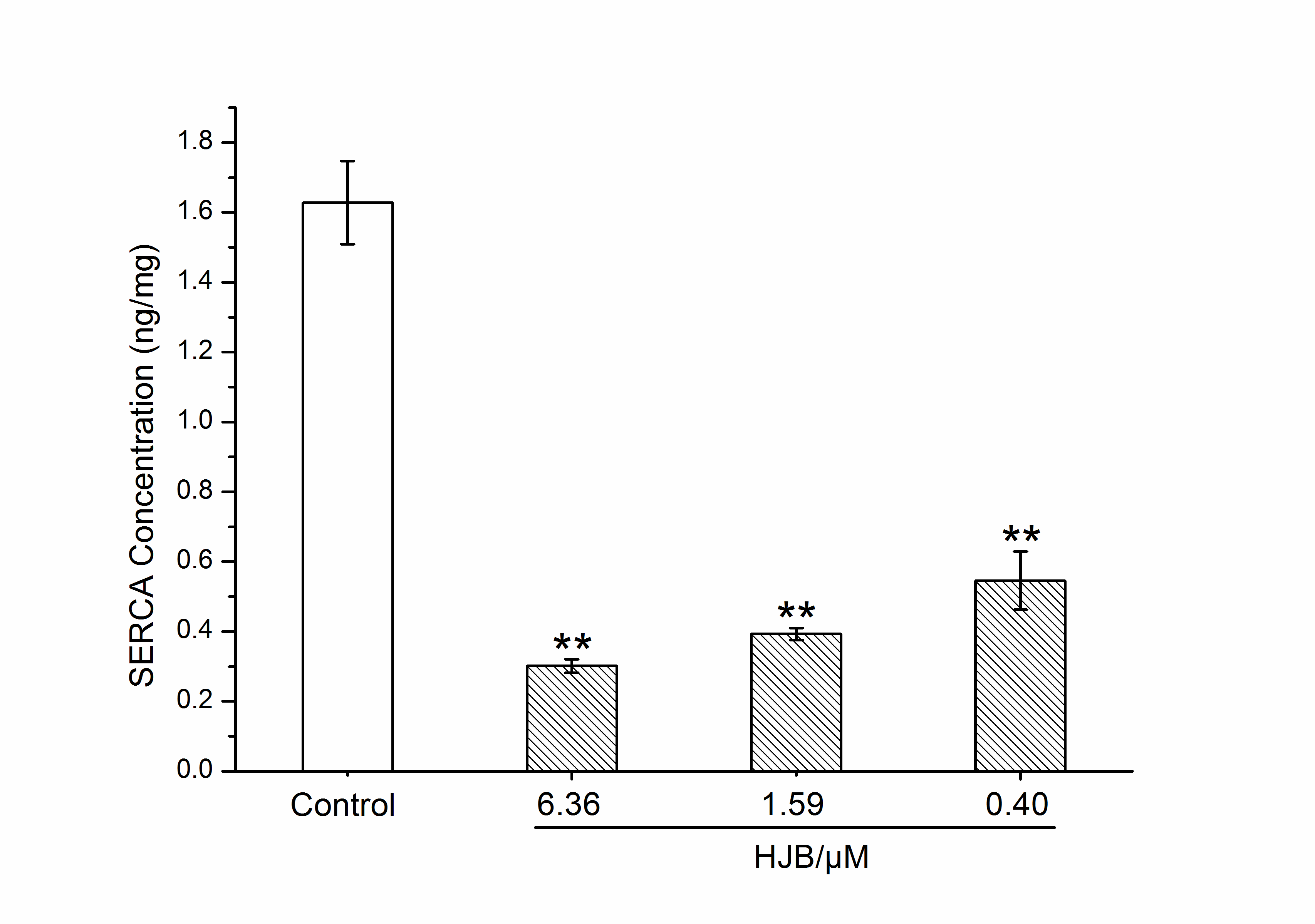

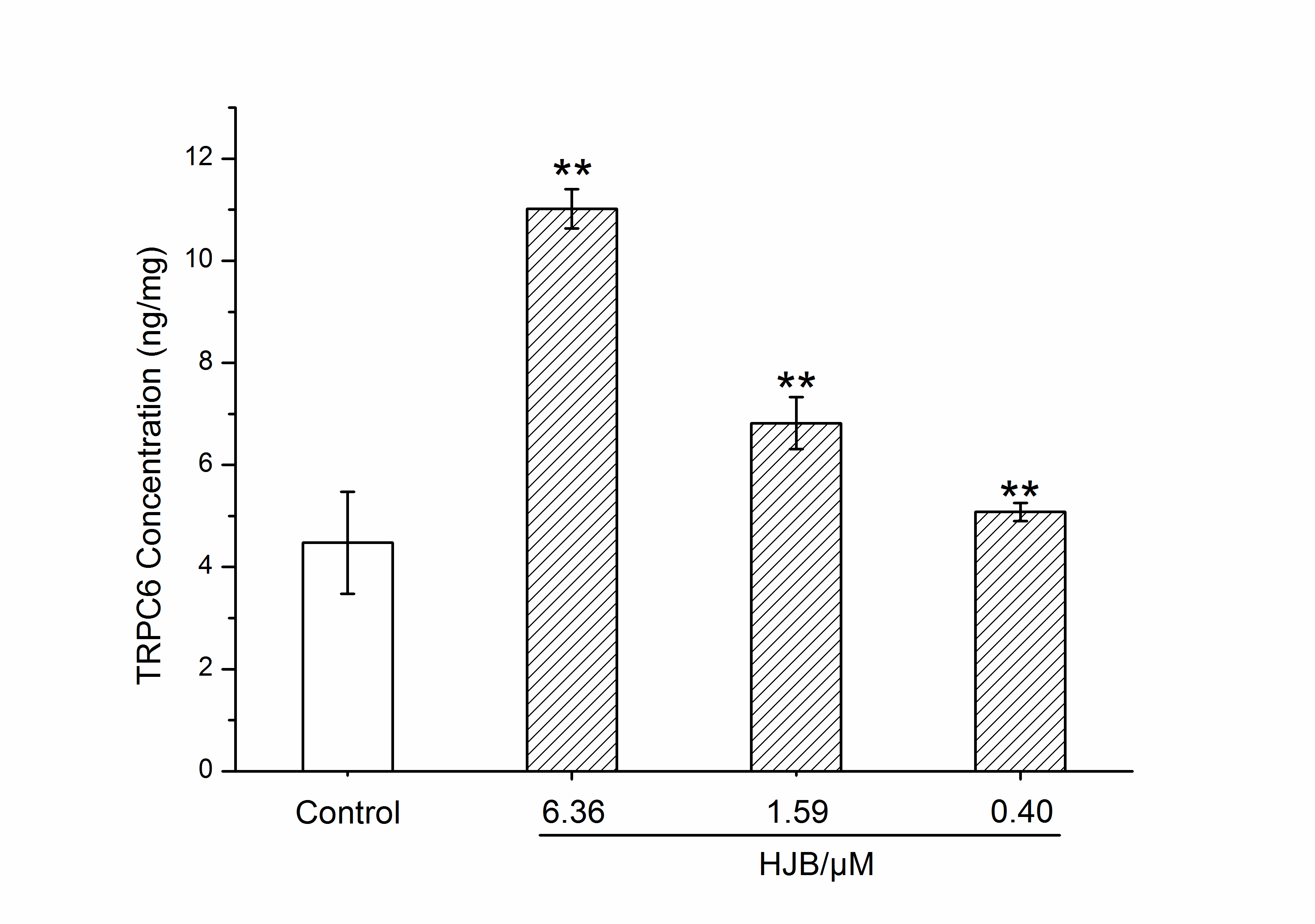


**Figure S9** Surface expression level of serca and TRPC6 in HL60 cells treated with different concentrations of HJB for 24 h. ^*^*p* < 0.05, ^**^*p* < 0.01 vs. control group. The results are representative of three independent experiments.

**Table S1 Matrix effects, recoveries, intra- and inter-day precisions and accuracies of HJB in rat plasma (n=5).**

| Added  (ng/ml) | Matrix effect  (%) | Recovery  (%) | RSD  (%) | Intra-day precision | |  | Inter-day precision | |
| --- | --- | --- | --- | --- | --- | --- | --- | --- |
|  |  |  |  | Measured (ng/ml) | RSD (%) |  | Measured  (ng/ml) | RSD (%) |
| 2.00 | 92.4 | 85.8 | 4.3 | 1.90±0.09 | 2.9 |  | 1.88±0.18 | 6.8 |
| 50.0 | 91.8 | 88.2 | 4.7 | 48.7±2.0 | 3.2 |  | 50.6±3.2 | 4.6 |
| 400 | 90.5 | 105.6 | 6.8 | 407±21 | 4.0 |  | 412±29 | 4.9 |

***Supplementary References***

Li, A.C., Alton, D., Bryant, M.S., Shou, W.Z., 2005. Simultaneously quantifying parent drugs and screening for metabolites in plasma pharmacokinetic samples using selected reaction monitoring information-dependent acquisition on a QTrap instrument. Rapid communications in mass spectrometry : RCM 19, 1943-1950.
